# Hypoxia inducible factor HIF-1 promotes myeloid-derived suppressor cells accumulation through ENTPD2/CD39L1 in hepatocellular carcinoma

**DOI:** 10.1038/s41467-017-00530-7

**Published:** 2017-09-11

**Authors:** David Kung-Chun Chiu, Aki Pui-Wah Tse, Iris Ming-Jing Xu, Jane Di Cui, Robin Kit-Ho Lai, Lynna Lan Li, Hui-Yu Koh, Felice Ho-Ching Tsang, Larry Lai Wei, Chun-Ming Wong, Irene Oi-Lin Ng, Carmen Chak-Lui Wong

**Affiliations:** 1 Department of Pathology, The University of Hong Kong, Hong Kong, Hong Kong; 2 State Key Laboratory for Liver Research, The University of Hong Kong, Hong Kong, Hong Kong

## Abstract

Myeloid-derived suppressor cells (MDSCs) possess immunosuppressive activities, which allow cancers to escape immune surveillance and become non-responsive to immune checkpoints blockade. Here we report hypoxia as a cause of MDSC accumulation. Using hepatocellular carcinoma (HCC) as a cancer model, we show that hypoxia, through stabilization of hypoxia-inducible factor-1 (HIF-1), induces ectoenzyme, ectonucleoside triphosphate diphosphohydrolase 2 (ENTPD2/CD39L1), in cancer cells, causing its overexpression in HCC clinical specimens. Overexpression of ENTPD2 is found as a poor prognostic indicator for HCC. Mechanistically, we demonstrate that ENTPD2 converts extracellular ATP to 5′-AMP, which prevents the differentiation of MDSCs and therefore promotes the maintenance of MDSCs. We further find that ENTPD2 inhibition is able to mitigate cancer growth and enhance the efficiency and efficacy of immune checkpoint inhibitors. Our data suggest that ENTPD2 may be a good prognostic marker and therapeutic target for cancer patients, especially those receiving immune therapy.

## Abstract (web summary)

Myeloid-derived suppressor cells (MDSCs) promote tumor immune escape. Here, the authors show that in hepatocellular carcinoma, hypoxia induces the expression of ENTPD2 on cancer cells leading to elevated extracellular 5′-AMP, which in turn promote the maintenance of MDSCs by preventing their differentiation.

## Introduction

Avoiding immune destruction represents a new hallmark of cancer. This process is closely associated with the presence of immune suppressive cells such as myeloid-derived suppressor cells (MDSCs) and regulatory T (Treg) cells within the tumor stroma. Hepatocellular carcinoma (HCC), primary liver cancer, is usually preceded by liver damage and extensive inflammation, and therefore is accompanied by infiltration of immune cells. How multiple immune populations are maintained in HCC remains largely elusive. Increase of MDSCs was found in the blood and tumors of HCC patients and in mice that bear HCC^1, 2^. MDSCs in HCC were able to inhibit T and natural killer (NK) cells, and activate Treg cells^3, 4^. MDSCs are bone marrow-derived myeloid progenitors. Human MDSCs are classified as CD11b^+^CD33^+^HLA-DR^−^, which may co-express with other markers such as CD15, CD14, CD115, and/or CD124^5^. Mouse MDSCs are classified as CD11b^+^Gr1^+^ and could be further sub-divided into the monocytic (M)-CD11b^+^Ly6C^+^Ly6G^−^ population and the polymorphonuclear (PMN)-CD11b^+^Ly6G^+^Ly6C^lo^ population^5^. MDSCs represent 30% of cells in the bone marrow and 2–4% cells in the spleen in normal mice. MDSCs normally differentiate into granulocytes, macrophages, or dendritic cells^5, 6^. However, under pathological conditions such as cancer, MDSCs become activated, maintain undifferentiation, and rapidly expand^5, 6^. In addition to T and NK cells, MDSCs also suppress dendritic cells. The broad immunosuppressive effects of MDSCs allow cancer cells to bypass immune surveillance^5, 6^. More importantly, MDSCs reduce T-cell infiltration into tumor and hence greatly reduce the clinical benefits of immune checkpoint therapies^7^. MDSCs also produce high levels of matrix metalloproteinase 9 (MMP9), which releases angiogenic factor, vascular endothelial growth factor, from the extracellular matrix, to promote growth of blood vessels^8^. A recent study showed that MDSCs maintain stemness properties of ovarian cancer cells^9^.

Hypoxia, oxygen (O_2_) deprivation, is an important environmental factor in HCC. The median O_2_ partial pressure in human HCC is 6 mm Hg as compared with 30 mm Hg in normal liver^10^. Regions of HCC frequently receive insufficient O_2_ supply as growth of HCC cells often exceeds growth of functional blood vessels. Common palliative HCC therapies, hepatic artery ligation (HAL), and transcatheter arterial (chemo) embolization (TAE/TACE), which initially intend to restrict HCC growth through blood (nutrient) supply obstruction, undesirably induce hypoxia. The major molecular mechanism elicit by hypoxia is through the stabilization of hypoxia-inducible factors (HIFs). HIFs are heterodimers consisting of an O_2_-sensitive HIF-1/2α subunit and a constitutively expressed HIF-1β subunit^11^. With O_2_ as the co-substrate, HIF-1/2α subunit is hydroxylated by prolyl hydroxylases (PHDs)^12^, allowing the recognition of von Hippel–Lindau protein (VHL) for ubiquitin-mediated proteosomal degradation of HIF-1/2α^13^. Decline of O_2_ stabilizes HIF-1/2α, which binds to HIF-1β, to initiate transcription of their target genes^14^. HIF-1/2α is highly expressed in HCC and is closely associated with poor clinical outcome in HCC patients. Inhibition of HIF-1α by oligonucleotides markedly enhanced the efficiency and efficacy of TACE in preclinical animal study^15^. HIFs, through inducing various chemokines, tightly orchestrate the immune context of tumor. HIFs promote Treg infiltration through chemokine (C–C motif) ligand 28 (CCL28) in ovarian cancer model^16^. We demonstrated in HCC model that hypoxic cancer cells recruit CX_3_CR1-expressing MDSCs to the tumor through chemokine (C–C motif) ligand 26 (CCL26)^17^. We further showed that obstructing the HCC–MDSC cell communication through targeting CCL26/CX_3_CR1 efficiently retarded HCC progression^17^.

Apart from chemokine induction, hypoxia stimulates transport of ATP into the extracellular space, which has significant impact on the tumor microenvironment. Extracellular ATP is hydrolyzed to 5′-AMP by ectonucleoside triphosphate diphosphohydrolase (ENTPD1, CD39), whereas extracellular 5′-AMP is further hydrolyzed to adenosine by 5′-nucleotidase (NT5E, CD73). These extracellular metabolites are known to tightly regulate neurotransmission and immune responses through interacting with the purigenic (G-coupled) receptors P2 and P1. ATP acts on the P2 receptor, whereas 5′-AMP and adenosine act on the P1 receptor of immune cells^18, 19^. Stimulation of P2 or P1 in immune cells results in very different biological responses, in general, extracellular ATP activates, whereas adenosine suppresses the immune system^19^. Extracellular ATP induces the differentiation of T cells and promotes the chemotaxis of macrophages and neutrophils^20^. Extracellular adenosine hampers the cytotoxicity of T and NK cells, and activates immune suppressive cells including MDSCs and Treg cells^20^. Although the roles of extracellular 5′-AMP are less documented, 5′-AMP is believed to share similar functions as adenosine due to the same receptors they stimulate^20^. Expression of ENTPD1 and NT5E have been shown to be HIF-1α dependent in epithelial cells^19^. In cancers, NTPD1 and NT5E, through creating an adenosine-rich tumor microenvironment, enable immune cells to escape immune surveillance and promote tumor survival^19^. ENTPD inhibitor, through suppressing ATP conversion to 5′-AMP, restores the recruitment of T and dendritic cells, and enhances the efficiency of chemotherapeutic agents in fibrosarcoma mouse model^21^.

Including ENTPD1, there are eight members in the ENTPD family and four of which have extracellular facing catalytic domain (ENTPD 1, 2, 3, and 8), but only the roles of ENTPD1 in cancer has been briefly reported^22^. Strikingly, we find that ENTPD2, but not the other ENTPD family members and NT5E, is dramatically overexpressed in HCC. We further show that hypoxia upregulates ENTPD2 through HIF-1α in HCC and subsequently leading to the accumulation of extracellular 5′-AMP, which maintains MDSCs undifferentiated. Blocking ENTPD2 in cancer cells remarkably restrained HCC growth in vivo. More excitingly, depletion of MDSCs through ENTPD2 inhibitor significantly improves the efficiency and efficacy of immune checkpoint inhibitors in vivo. Our study brings three important messages. First, we disclose a novel mechanism by which hypoxia/HIF-1α shapes the tumor microenvironment. Second, we highlight how ENTPD2 is harnessed by cancer cells to escape immune-mediated destruction. Third, we reveal a novel therapeutic approach that shows great synergism with immunotherapies in HCC, a second most deadly cancer which to date has no promising curative treatments.

## Results

### ENTPD2 is predominantly expressed under hypoxia in human HCC

**Fig. 1:**
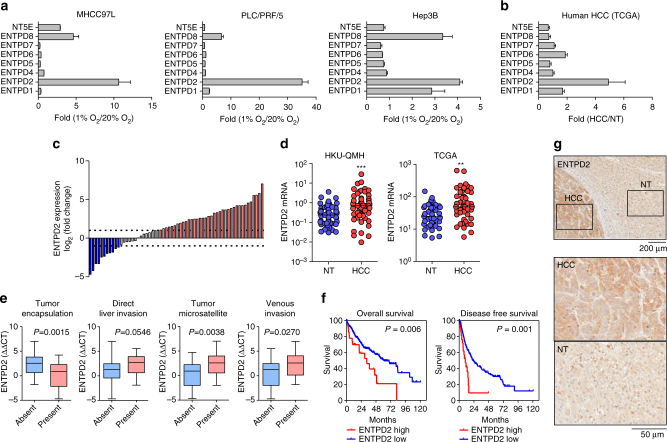
ENTPD2 expression in HCC. **a** Real-time quantitative PCR analysis revealed the expression of ENTPD family members and NT5E in three HCC cell lines, MHCC97L, PLC/PRF/5, and Hep3B, exposed to 20 and 1% O_2_. Gene expression level was normalized to 20% O_2_ (*n* = 3). **b** TCGA database revealed the expression of ENTPD family members and NT5E in 49 cases of HCC tissues and their corresponding non-tumorous liver (NT) tissues. Gene expression level was normalized to NT. **c** Waterfall plot showed that ENTPD2 was overexpressed in 58% (36/62) of HCC patients. **d** *Left*: ENTPD2 mRNA expression in HCC tissues and their corresponding NT tissues from 62 patients = (Queen Mary Hospital (QMH), the University of Hong Kong (HKU)) was determined by qRT-PCR. *Right*: ENTPD2 mRNA expression in HCC and NT tissues in 49 HCC patients from the TCGC database. For qRT-PCR, values were normalized with house-keeping gene, *HPRT*. (Wilcoxon signed-rank test, **P < 0.01, ***P < 0.001) **e** High expression of ENTPD2 was associated with aggressive clinico-pathological features (*n* = 62, Student’s *t*-test). **f** Data from TCGA showed that high expression (*z*-score > 1) of ENTPD2 was significantly associated with poorer overall and disease free survival in 368 and 347 HCC patients, respectively (log-rank test, *P* < 0.01). **g** Representative IHC staining images of ENTPD2 in human HCC and NT tissues (10 cases). High ENTPD2 staining was only observed in HCC tissues but not in NT tissues

We and others have previously shown that MDSCs represent an important tumor-residing immune population. Interestingly, we used HCC as a cancer model to show that MDSCs favorably infiltrate into hypoxic regions of tumors^17^. We have further demonstrated that MDSCs are attracted to tumors through CCL26, whose expression is tightly orchestrated by HIFs^17^. This interesting finding has provided a partial mechanistic explanation for the intimate relationship of hypoxia and MDSC. As MDSCs are prone to differentiation, how intra-tumoral hypoxia maintains MDSC in undifferentiated status upon their arrival demands greater understanding. To comprehensively parse the roles of hypoxia in MDSC accumulation in tumors, we investigated the ENTPD family and NT5E, which have been currently demonstrated as the important contributors in the immunosuppressive tumor microenvironment^23^. From real-time quantitative PCR analysis of three HCC cell lines, MHCC97L, PLC/PRF/5, and Hep3B, exposed to 20% O_2_ (normoxia) and 1% O_2_ (hypoxia), it was observed that several members in ENTPD family or NT5E were induced by hypoxia with ENTPD2 achieving the greatest upregulation consistently in three HCC cell lines (Fig. 1a and Supplementary Fig. 1a). We also retrieved data from the TCGA (The Cancer Genome Atlas) in 49 pairs of human HCC tissues and their corresponding non-tumorous (NT) liver tissues. Clinically, ENTPD2 was also predominantly overexpressed in HCC patients (Fig. 1b), implying that ENTPD2 might have a unique role in promoting tumorigenesis. Overexpression of ENTPD2 messenger RNA was further validated in an expanded cohort of 62 HCC patients. TCGA data showed that ENTPD2 was consistently upregulated in an independent cohort of HCC patients (Fig. 1d). More excitingly, ENTPD2 upregulation was associated with several aggressive HCC clinico-pathological features including presences of direct liver invasion, tumor microsatellite formation and venous invasion, as well as the absence of tumor encapsulation (Fig. 1e). High ENTPD2 expression was also significantly correlated with poor disease-free and overall survival in HCC patients (Fig. 1f). Immunohistochemistry staining in human HCC cases confirmed that ENTPD2 was also overexpressed in protein level in human HCCs relative to their NT counterparts (Fig. 1g) and its staining followed a typical oxygen diffusion pattern (Supplementary Fig. 1b) as we reported previously^24^, suggesting that ENTPD2 is tightly regulated by hypoxia.

### ENTPD2 is a direct transcriptional target of HIF-1α

**Fig. 2:**
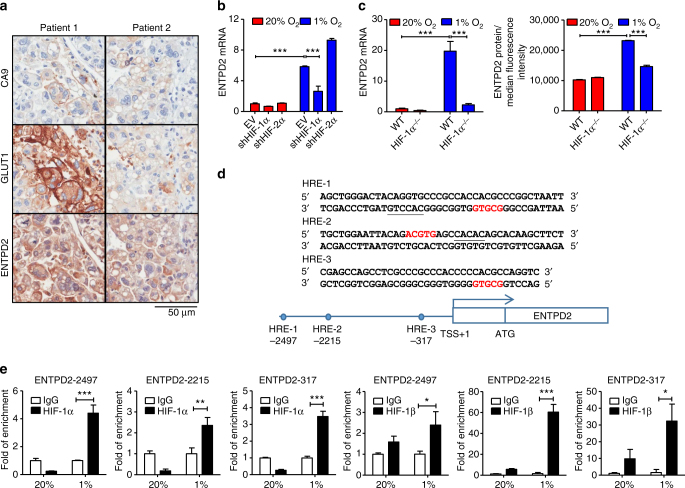
ENTPD2 is a direct transcriptional target of HIF-1α. **a** Representative IHC staining images of ENTPD2 and two hypoxia markers, CA9 and GLUT1, in human HCC. Overlapping staining pattern of ENTPD2, CA9, and GLUT1 was observed. **b** ENTPD2 mRNA expression in MHCC97L-EV, -shHIF-1α, and shHIF-2α clones. Cells were exposed to 20 and 1% O_2_ for 24 h and ENTPD2 mRNA expression was quantitated by qRT-PCR. Values were normalized to 20% O_2_ EV or WT (*n* = 3). **c** ENTPD2 mRNA (*left*) and protein (*right*) expression in MHCC97L- HIF-1α-WT (parental) and HIF-1α-knockout (HIF-1α−/−) clones. Cells were exposed to 20 and 1% O_2_ for 24 h. The mRNA and protein expressions of ENTPD2 were quantitated by qRT-PCR and flow cytometry, respectively. All values were normalized to 20% O_2_ WT (*n* = 3). **d** Three putative hypoxia-responsive elements (HREs), HIF binding consensus sequence, 5′-A/GCGTG-3′ in the promoter region of ENTPD2 were located by in silico analysis. **e** ChIP assay was performed in MHCC97L cells exposed to 20 and 1% O_2_ with IgG, HIF-1α, and HIF-1β antibodies. Fold of enrichment was normalized to the according IgG controls (*n* = 3). Data are presented as mean ± s.d. (Student’s *t*-test, ***P < 0.001)

To provide additional evidence that ENTPD2 was regulated by hypoxia, we stained consecutive sections of 10 cases of human HCCs with ENTPD2 and 2 hypoxia markers including glucose transporter 1 (GLUT1) and carbonic anhydrase IX (CA9), and observed a significant overlapping staining pattern (Fig. 2a). ENTPD1 has been demonstrated to be regulated by HIF-1α^25^. To confirm whether ENTPD2 is regulated by HIFs, we examined ENTPD2 mRNA expression in HIFs knockdown and knockout (KO) HCC cells. We found that the hypoxia-induced ENTPD2 expression could be abrogated upon knockdown or KO of HIF-1α, but not HIF-2α in MHCC97L cells (Fig. 2b, c and Supplementary Fig. 2). To confirm whether ENTPD2 is a direct transcriptional target of HIF-1α, three putative hypoxia-responsive elements (HREs) containing HIF-1α-binding consensus sequence 5′-A/GCGTG-3′ in the promoter region of ENTPD2 were located. Chromatin immunoprecipitation (ChIP) assay demonstrated the enrichment of hypoxia-inducible binding of HIF-1α and HIF-1β to these putative HREs in ENTPD2 (Fig. 2d, e).

### ENTPD2 promotes HCC tumor growth and MDSC accumulation

**Fig. 3:**
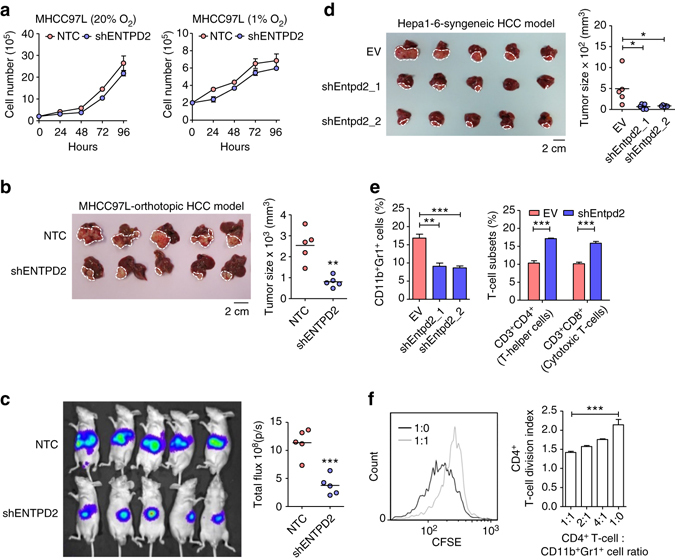
ENTPD2 promotes tumor growth and MDSC accumulation. **a** Proliferation rate of MHCC97L-NTC and -shENTPD2 clones in 20 and 1% O_2_ (*n* = 3). BALB/c nude mice were orthotopically implanted with 1 × 10^6^ MHCC97L-NTC, -shENTPD2 clones (*n* = 5 for each groups). Tumor size was measured with **b** caliper and **c** Xenogen imaging. C57BL/6 mice were orthotopically implanted with 3 × 10^6^ Hepa1-6-EV, -shEntpd2_1, and -shEntpd2_2 clones (*n* = 5 for each group). **d** Tumor size was measured with caliper. **e** Tumors collected were dissociated. The percentages of CD11b^+^Gr1^+^ cells, CD3^+^CD4^+^ cells, and CD3^+^CD8^+^ cells in the tumors were determined by flow cytometry (*n* = 3). **f** CD11b^+^Gr1^+^ cells were isolated from tumor by magnetic cell sorting and co-cultured with CFSE-labeled splenic CD3^+^CD4^+^ cells at different T cell : MDSC ratios. The fluorescence intensity of CFSE for each cell would halve after cell division. Cell proliferation of T cells was indicated by reduction of CFSE intensity as a result of cell division. The proliferation index of CD3^+^CD4^+^ cells was analyzed by flow cytometry (*n* = 3). Data are presented as mean ± s.d. (Student’s *t*-test, *P < 0.05, **P < 0.01, ***P < 0.001)

To evaluate the impact of ENTPD2 on HCC tumorigenesis, we established ENTPD2 knockdown clones by short hairpin (sh) RNA approach in two HCC cell lines, MHCC97L (human) and Hepa1-6 (mouse), without altering the expression of other ENTPD family members (Supplementary Fig. 3). ENTPD2 had no direct effects on HCC cell proliferation in vitro (Fig. 3a and Supplementary Fig. 4). To evaluate the effects of ENTPD2 in HCC growth in vivo, we orthotopically inoculated the MHCC97L subclones into BALB/c nude mice and found that knockdown of ENTPD2 drastically reduced tumor growth (Fig. 3b, c). As BALB/c nude mice lack T cells and therefore this model might not be able to fully reflect the immunosuppressive roles of MDSCs, we also performed orthotopic implantation with ENTPD2 knockdown Hepa1-6 cells in immune-competent C57BL/6. Notably, ENTPD2 was also upregulated under hypoxia in mouse HCC cell lines (Supplementary Fig. 5). As Hepa1-6 was derived from C57BL/6 mice, we found no immune rejection in this syngeneic model. Concordantly, knockdown of ENTPD2 in mouse HCC cells drastically repressed tumor growth in immune-competent mice (Fig. 3d). The disparate effects of ENTPD2 on in vitro and in vivo cell growth implied that ENTPD2 might promote tumor growth through shaping the tumor microenvironment rather than having direct impact on tumor cell itself. Interestingly, although ENTPD1 and ENTPD2 share similar functions, demonstrated by ENTPD1- and ENTPD2-KO tumor models, ENTPD2 had greater effects on tumor growth, implying that ENTPD2 took the major role in HCC progression (Supplementary Figs. 6–7). Next, we proceeded to evaluate the percentages of various immune populations in the tumors derived from ENTPD2 knockdown and control cells. Excitingly, we found that knockdown of ENTPD2 in HCC cells reduced the number of MDSCs in the tumors as represented by CD11b^+^Gr^+^ cell population, but increased the number of CD4^+^ and CD8^+^ T-cell populations (Fig. 3e). We further isolated CD11b^+^Gr^+^ cells from the tumors and co-cultured them with carboxyfluorescein succinimidyl ester (CFSE)-labeled CD4^+^ T cells. The fluorescence intensity of CFSE for each cell would be halved after cell division. In other words, cell proliferation can be indicated by halving of the fluorescence intensity of CFSE. CD11b^+^Gr^+^ cells suppressed the proliferation of CD4^+^ T cells, indicating that CD11b^+^Gr^+^ cells in our tumor models possessed immunosuppressive activity and are functionally considered as MDSCs (Fig. 3f). Taken together, these data converged to support that ENTPD2 promoted HCC tumorigenesis and MDSC accumulation.

### ENTPD2 supports MDSC maintenance through 5′-AMP production

**Fig. 4:**
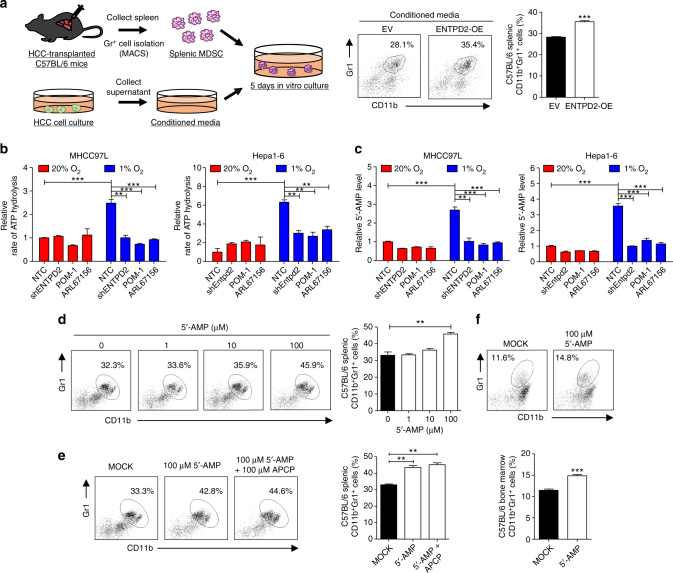
ENTPD2 hydrolyzes ATP to 5′-AMP that contributes to MDSC maintenance. **a** The effect of ENTPD2 on MDSC maintenance. Splenic MDSCs isolated from C57BL/6 mice were cultured in conditioned media collected from MHCC97L-EV and -ENTPD2-overexpressing (OE) clones. The percentages of CD11b^+^Gr1^+^ cells after 4-day culturing were analyzed (*n* = 3). **b** Liquid chromatography-mass spectrometry (LC-MS) analysis of the rate of ATP hydrolysis in conditioned media (*n* = 3). **c** Liquid chromatography-mass spectrometry (LC-MS) analysis of adenosine monophosphate (5′-AMP) level in conditioned media. MHCC97L and Hepa1-6 cells were pre-exposed to 20% O_2_ and 1% O_2_ for 48 h. Cells were trypsinized and 2 × 10^6^ cells were re-suspended in serum-free DMEM medium supplemented with 100 µM ATP and incubated at 37 °C for 1 h. Conditioned media were collected and subjected to LC-MS analysis (*n* = 3). **d**–**f** The effect of 5′-AMP on MDSC maintenance. Splenic CD11b^+^Gr1^+^ cells isolated from C57BL/6 mice were cultured in the presence of **d** different concentrations of 5′-AMP and **e** 100 µM 5′-AMP with or without NT5E inhibitor, APCP. The percentages of CD11b^+^Gr1^+^ cells after 4-day culturing were analyzed (*n* = 3). **f** The effect of 5′-AMP on MDSC induction from bone marrow progenitors. Bone marrow progenitors collected from C57BL/6 mice were cultured with GM-CSF and IL4 with or without 5′-AMP. The percentages of CD11b^+^Gr1^+^ cells induced after 4-day culturing were analyzed (*n* = 3). Data are presented as mean ± s.d. (Student’s *t*-test, *P < 0.05, **P < 0.01, ***P < 0.001)

To thoroughly investigate how ENTPD2 promoted MDSC accumulation, we stably overexpressed ENTPD2 using the CRISPR-dCas9 synergistic activator system (SAM) system in HCC cells (Supplementary Fig. 8a, b) and collected the conditioned media to culture the freshly isolated splenic CD11b^+^Gr1^+^ MDSCs from C57BL/6 mice. More CD11b^+^Gr1^+^ MDSCs were maintained when cultured in conditioned medium (CM) collected from ENTPD2-overexpressing HCC cells as compared with empty vector (EV) control (Fig. 4a). As the function of ENTPD2 is to dephosphorylate extracellular ATP into ADP and subsequently into 5′-AMP, we examined the effect of ENTPD2 on the levels of these extracellular metabolites in HCC cells. Liquid chromatography-mass spectrometry (LC-MS) analysis showed that hypoxia increased the rate of extracellular ATP hydrolysis and the amount of extracellular 5′-AMP in the CM of both human (MHCC97L) and mouse (Hepa1-6) HCC cell lines. Meanwhile, those effects were abrogated upon knockdown and KO of ENTPD2 or inhibition of ENTPD2 by two ENTPD2 inhibitors, ARL67156 and POM-1. These data indicated that ENTPD2 of cancer cells promoted the conversion of extracellular ATP to 5′-AMP in the microenvironment (Fig. 4b, c and Supplementary Fig. 9). To test whether ENTPD2 regulates MDSCs through 5′-AMP, we cultured CD11b^+^Gr1^+^ MDSCs in the presence of different concentrations of 5′-AMP. Levels of 5′-AMP positively correlated with the amount of CD11b^+^Gr1^+^ MDSCs remaining in culture after 4 days (Fig. 4d and Supplementary Fig. 10). It has been reported that MDSCs might express NT5E, which can further dephosphorylate extracellular 5′-AMP into adenosine that might also promote MDSC accumulation^26^. However, LC-MS analysis showed that the level of adenosine in the MDSC culture was undetectable (Supplementary Fig. 11). We further showed that the presence of competitive inhibitor of NT5E, APCP, could not abrogate the effect of 5′-AMP on MDSC maintenance (Fig. 4e and Supplementary Fig. 11). Furthermore, we demonstrated that 5′-AMP could increase the amount of CD11b^+^Gr1^+^ MDSCs induced from bone marrow progenitors (Fig. 4f). These suggested that 5′-AMP is the key extracellular metabolite that modulates MDSCs in our experimental model.

### ENTPD2 and 5′-AMP regulate fate of M-MDSC differentiation

**Fig. 5:**
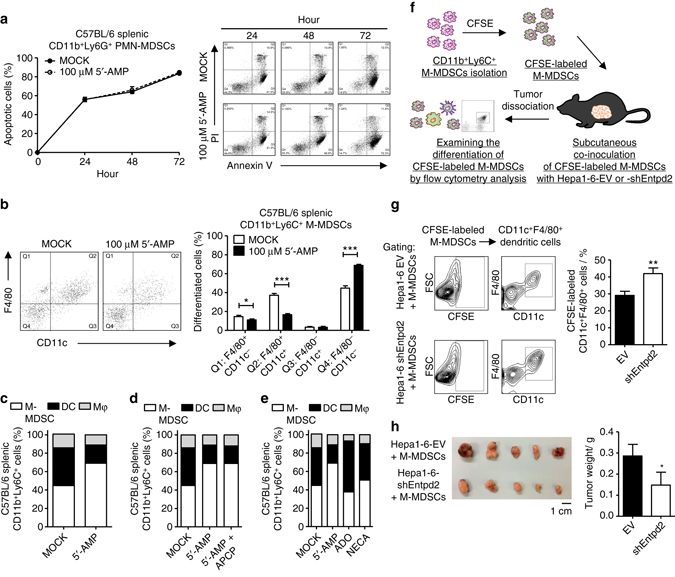
5′-AMP impairs M-MDSC differentiation to dendritic cells. **a** The effect of 5′-AMP on apoptotic rate of PMN-MDSCs. Splenic CD11b^+^Ly6G^+^ cells isolated from C57BL/6 mice were cultured with or without 100 µM 5′-AMP for annexin V assay at different time points (*n* = 3). The effect of 5′-AMP or adenosine on M-MDSC differentiation. Splenic CD11b^+^Ly6C^+^ cells isolated from C57BL/6 mice were cultured for 4 days **b**, **c** with or without 100 µM 5′-AMP, **d** with 100 µM 5′-AMP in the presence or absence of NT5E inhibitor, APCP, or **e** with 100 µM 5′-AMP, 100 µM adenosine (ADO), 100 µM 5′-APCP, or 100 µM NECA. Expression for two surface markers, CD11c and F4/80, were analyzed by flow cytometry and the distribution of CD11c^−^F4/80^+^ macrophage (Mφ), CD11c^+^F4/80^−^ and CD11c^+^F4/80^+^ dendritic cell (DC) and CD11c^−^F4/80^−^ M-MDSC populations were determined by the surface markers (*n* = 3). **f** The effect of ENTPD2 on the fate of M-MDSCs in vivo. Splenic CD11b^+^Ly6C^+^ cells isolated from C57BL/6 mice were stained with fluorescent cell tracking dye CFSE and co-inoculated with Hepa1-6-EV or shEntpd2 clones into C57BL/6 mice subcutaneously (*n* = 5 for each groups). **g** The tumors were dissociated and stained with anti-CD11c and anti-F4/80 to examine the differentiation of CFSE-labelled M-MDSCs (*n* = 5 for each groups). **h** Tumor weights were measured (*n* = 5 for each groups). Data are presented as mean ± s.d. (Student’s *t*-test, *P < 0.05, **P < 0.01, ***P < 0.001)

CD11b^+^Gr1^+^ MDSCs consist of two subsets, CD11b^+^Ly6C^+^ M-MDSCs and CD11b^+^Ly6G^+^ PMN-MDSCs. During culture, M-MDSCs tend to differentiate into dendritic cells or macrophages, whereas PMN-MDSCs fail to differentiate and undergo apoptosis^27, 28^. To investigate how 5′-AMP supported MDSC maintenance, we isolated the two MDSCs subsets from C57BL/6 mice and examined their individual responses to 5′-AMP. 5′-AMP did not affect the survival of PMN-MDSC and total MDSCs (Fig. 5a and Supplementary Fig. 12). However, we clearly observed that less M-MDSCs acquired the mature markers, F4/80 and CD11c, in the presence of 5′-AMP (Fig. 5b, c and Supplementary Fig. 13a, b). CD11b^+^Ly6C^+^F4/80^+^CD11c^+^ cells can be identified as monocyte-derived dendritic cells^29^. This suggested that 5′-AMP prevented M-MDSC from maturation. As APCP could not reverse the phenotype (Fig. 5d), this suggested the effect is mainly associated with 5′-AMP but not adenosine. To further support this argument, we compared the effects of 5′-AMP, adenosine, and non-selective adenosine receptor agonist, 5′-*N*-ethylcarboxamidoadenosine (NECA), on M-MDSC maturation. Adenosine had no effect, whereas NECA only had mild effects (Fig. 5e). Next, to investigate whether ENTPD2 could regulate the fate of M-MDSC differentiation in vivo, CFSE-labeled M-MDSCs were subcutaneously co-inoculated with Hepa1-6-EV or shEntpd2 clones into C57BL/6 mice (Fig. 5f). Tumors were dissociated and the status of CSFE-labeled cells was examined by staining dendritic cell surface markers followed by flow cytometry analysis (Fig. 5g). We found that knockdown of ENTPD2 led to an increase of CFSE-labeled CD11c^+^ F4/80^+^ population (Fig. 5g), suggesting that ENTPD2 prevents M-MDSCs and total MDSCs from differentiating into dendritic cells. Co-inoculation of M-MDSCs with ENTPD2 knockdown HCC cells also repressed tumor growth as compared with control HCC cells (Fig. 5h). Consistent results were observed in nude mice (Supplementary Fig. 13c).

### ENTPD2 inhibitor increases efficacy of anti-PD-1/CTLA-4

**Fig. 6:**
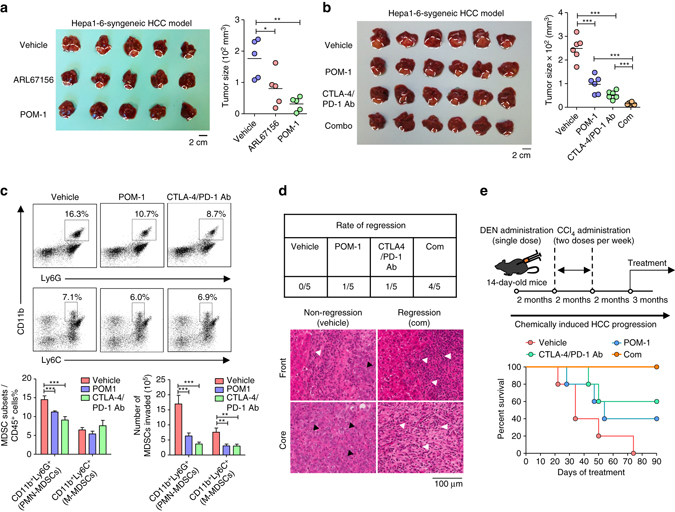
Combined treatment with ENTPD2 inhibitor and anti-PD-1/anti-CTLA-4 immune checkpoint inhibitors in C57BL/6 HCC-bearing mice. **a** C57BL/6 mice were orthotopically implanted with 3 × 10^6^ Hepa1-6. On day 3, mice were administered with vehicle, 3 mg kg^−1^ ARL67156 or 10 mg kg^−1^ POM-1 through i.p. injection for 8 consecutive days (*n* = 5 for each group). Images of tumors collected from mice and tumor size was measured with caliper. **b** C57BL/6 mice were orthotopically implanted with 3 × 10^6^ Hepa1-6. On day 3, mice were administered with vehicle or 10 mg kg^−1^ POM-1 i.p. injection for 8 consecutive days, whereas on day 5 and day 9, mice were administered with 4 mg kg^−1^ control IgG antibody or 2 mg kg^−1^ anti-PD-1 and anti-CTLA-4 immune checkpoint inhibitors (CTLA-4/PD-1 Ab) through i.p. injection (*n* = 6 for each group). Images of tumors collected from mice and tumor size was measured with caliper. **c** Tumors collected at Day 11 were dissociated and the percentages of CD11b^+^Ly6C^+^ M-MDSCs and CD11b^+^Ly6G^+^ PMN-MDSCs in CD45^+^ cell population and the total number of MDSCs were determined by flow cytometry. **d** Tumors collected at Day 18 were sectioned and stained with hematoxylin and eosin for histological analysis (*n* = 5 for each group). The representative pictures of tumor boundaries (front) and tumor cores (core) of vehicle and combined treatment group were shown. *Black arrows* indicated the tumor cells. *White arrows* indicated the immune cells. **e** Survival test of chemically-induced HCC mice. Two-week-old C57BL/6 mice were administered with single dose of 1 mg kg^−1^ DEN through i.p. injection (Sigma-Aldrich) and after 8 weeks, with 1.25 ml kg^−1^ 30% CCl_4_ (Sigma-Aldrich) through i.p. injection twice weekly for 8 weeks. Twenty-six-week-old mice were with administered 10 mg kg^−1^ POM-1 through i.p. injection thrice weekly or 2 mg kg^−1^ PD-1and CTLA-4 monoclonal antibodies through i.p. injection twice weekly (*n* = 5 for each group). Data are presented as mean ± s.d. (Student’s *t*-test, *P < 0.05, **P < 0.01, ***P < 0.001)

As MDSCs could suppress T cells, the presence of MDSCs is often associated with decrease of T-cell infiltration in tumors, thereby reducing the efficiency and efficacy of immunotherapies that involve the activation of T cells. CTLA-4 (cytotoxic T lymphocyte-associated protein 4) and PD-1 (programmed cell death receptor 1) are the most hotly pursued and widely studied immune checkpoint receptors for immunotherapies in cancers^30^. CTLA-4 and PD-1 are inhibitory receptors of T cells^30^. CTLA-4 prevents activation of T cells by competing with CD28 (stimulatory T-cell receptor) for their ligands CD80 and CD86^30^. PD-1, upon stimulation by its ligands PD-L1 and PD-L2, which are often expressed in cancer cells, inhibits kinases involved in T-cell activation^30^. Anti-CTLA-4 antibody (Ipilimumab) and anti-PD-1 antibody (Nivolumab) were first approved by Food and Drug Administration for the treatment of melanoma and have remarkable long-term survival benefits in cancer patients, whereas their efficiency and efficacy in HCC patients are still unknown^30^. We hypothesize that blockade of ENTPD2 in cancer cells would impair MDSC maintenance and enhance T-cell penetrance in the tumors, thereby improving the effectiveness of immune checkpoint therapies in cancer treatment. Single treatment of ENTPD2 inhibitors, ARL67156 and POM-1, markedly reduced growth of Hepa1-6-derived HCC in C57BL/6 syngeneic model (Fig. 6a). Notably, POM-1 has a slightly greater inhibitory effect on HCC as compared with ARL67156. POM-1 can inhibit both ENTPD1 and ENTPD2. To address whether the tumor inhibitory effects of POM-1 are mediated through ENTPD2, we inoculated Entpd1 KO HCC cells into C57BL/6 mice. We found that POM-1 could still significantly repress HCC growth in the tumors without Entpd1, suggesting that POM-1 inhibited HCC growth through Entpd2 in Hepa1-6 (Supplementary Fig. 14). As combined treatments of anti-CTLA-4 and anti-PD-1 antibodies have been demonstrated to be more effective in activating T cells within the tumors than single antibody treatment in mouse melanoma model^31^, as proof-of-concept we examined the effect of combined immune checkpoint inhibitors (anti-CTLA-4 and anti-PD-1) with POM-1. Combination of POM-1 and the two immune checkpoint inhibitors (Com) achieved the highest tumor suppressive responses (Fig. 6b). Tumor-associated MDSCs were significantly reduced by either POM-1 or immune checkpoint inhibitors treatment (Fig. 6c). Collecting the tumors at a longer time point, we found that the incidence of regression was significantly elevated by single treatments and, to even a greater extent, by combined treatment (Fig. 6d). Furthermore, combination of POM-1 and immune checkpoint inhibitors significantly increased infiltration of lymphocytes (white arrows) and reduced tumor cells (black arrows) (Fig. 6d). To study the effects of immune checkpoint inhibitors for an extended period of time, we employed a chemically induced mouse HCC models with nitrosodiethylamine (DEN) and carbon tetrachloride (CCl_4_). In this model, we further demonstrated combined treatment could maximize the survival of the HCC-bearing mice (Fig. 6e). Taken together, our study suggests inhibition of ENTPD2 as a promising strategy to increase the efficacy of PD-1/CTLA-4 immune checkpoint inhibitor in cancer treatment.

## Discussion

**Fig. 7:**
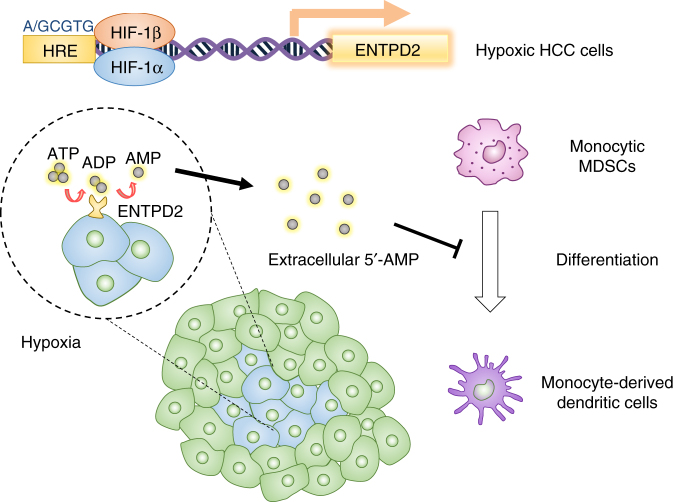
Schematic representation of hypoxia-induced MDSC accumulation. Hypoxia stabilizes HIF-1α in cancer cells. HIF-1α binds to the HRE in the promoter of ENTPD2 to initiate transcription in cancer cells. ENTPD2 facilitates the dephosphorylation of extracellular ATP into 5′-AMP. Increase in extracellular 5′-AMP prevents M-MDSCs from maturation at tumor sites, thereby promoting tumor-mediated immune escape. Inhibition of ENTPD2 reduces the number of tumor-infiltrating MDSCs and increase the efficacy of PD-1/CTLA-4 immune checkpoint inhibitors

The microenvironment of tumor is composed of various types of immune cells such as T cells, dendritic cells, and NK cells, which function to kill cancer cells. However, these immune cells are often exhausted or repressed by populations of immune suppressive cells such as Tregs and MDSCs leading to escape of immune surveillance of tumors. MDSCs are abundantly found in HCC^1, 2^. In general, there are at least two possible strategies to eradicate tumor-infiltrating MDSCs—preventing MDSC recruitment and promoting MDSC maturation^32^. Using HCC as a model, we previously demonstrated hypoxia as a central driver for MDSC accumulation in tumors and showed that hypoxic cancer cells secreted CCL26 to attract MDSCs^33^. Blockade of CCL26 cognate receptor, CX_3_CR1, by neutralizing antibody prevented MDSC infiltration and perturbed tumor growth. In this current study, we further revealed that hypoxia prevented MDSC maturation upon their arrival at tumor sites. We demonstrated that hypoxia induced ENTPD2 in cancer cells to generate a 5′-AMP-rich microenvironment to keep MDSC undifferentiated (Fig. 7).

Extracellular 5′-AMP is known to be catabolized into adenosine by ectoenzyme NT5E (CD73), which is widely expressed in endothelial cells, epithelial cells, several immune cells (mainly Treg cells and MDSCs), as well as tumor cells. Adenosine has an extensive impact on both lymphoid and myeloid lineages. For lymphocytes, adenosine was shown to decrease granule exocytosis-induced cytotoxicity and cytokine production of CD8^+^ effector T cells and to shift the polarization of CD4^+^ helper cells towards Tregs instead of Th1 cells^34–36^. For myeloid cells, adenosine was demonstrated to promote and maintain alternative macrophage activation via A_2B_ adenosine receptor so that M2 macrophages exhibit more potent immunosuppressive activity^37–39^. Notably, adenosine was also demonstrated to induce the expansion of PMN-MDSCs from bone-marrow myeloid progenitors and facilitate their ability to suppress T-cell proliferation with no observable effects on M-MDSCs^26^. Although adenosine and NT5E were extensively investigated in myeloid cells, the effects of 5′-AMP are largely unknown. Intriguingly, our data showed that ENTPD2 but not NT5E was significantly upregulated in HCC, further highlighting the importance of ENTPD2 and 5′-AMP in microenvironment formation and cancer development. More excitingly, we provided evidence showing that hypoxia induces ENTPD2 through HIF-1α. Mechanistically, we unambiguously showed that 5′-AMP prevented M-MDSCs from differentiating into dendritic cells in vitro and in vivo. In concordance with our clinical observation of ENTPD2 overexpression, we further showed that NT5E inhibitor could not prevent the 5′-AMP-induced MDSC maintenance, suggesting that ENTPD2 but not NT5E is critical for MDSC maintenance in the context of cancer.

MDSCs, especially M-MDSCs, can differentiate into either immune suppressive M2 macrophages or non-immune suppressive dendritic cells and M1 macrophages in tumors in the presence of specific cytokines. Stimulating the differentiation of MDSC into non-immune suppressive cells is an attractive anti-cancer strategy^32^. All-*trans* retinoic acid (ATRA), a metabolite of vitamin A, is a well-studied promoter of myeloid cell differentiation. ATRA can stimulate MDSCs into mature myeloid cells^40^ and also decrease their immunosuppressive activities via ERK1/2 kinase pathway;^41^ however, ATRA simultaneously induces the expansion of Treg cells^42^, another important immune suppressive population, precluding ATRA from being a suitable anti-cancer drug. Accumulative studies converged to show that hypoxia is a key factor, which determines the fate of tumor-infiltrating myeloid cell. A recent study showed that hypoxia increased sialic acid transport to cell membranes of MDSCs, promoting sialyation-mediated CD45 dimerization and therefore its activation. This led to downregulation of STAT3 activity, which consequently promoted the differentiation of M-MDSCs into tumor-associated macrophages (TAMs) or M2 macrophages^43^. Apart from being able to differentiate into TAMs as shown by others, our study has unambiguously indicated that hypoxia-induced ENTPD2 upregulation and subsequent 5′-AMP enrichment prevented the differentiation of M-MDSCs into non-immune suppressive CD11c^+^ dendritic cells in vivo. Both M-MDSCs and TAMs are immunosuppressive and pro-angiogenic, but M-MDSCs are maintained via 5′-AMP, whereas TAMs are maintained via adenosine^37–39^. The effects of 5′-AMP on alternative macrophage activation still remains to be addressed. Notably, our current study highlighted the mechanism by which hypoxia/HIF-1 in cancer cells signals MDSC. HIF-1 in MDSCs could regulate their intrinsic functions. HIF-1 itself or through its transcriptional target, microRNA-210, enhanced MDSC suppressive abilities by promoting their own arginase and nitric oxide production^44^. Our studies together with others have unraveled the multiple functions of hypoxia/HIF on MDSC in cancers and provided evidence that hypoxia is a critical modulator of the tumor stroma.

Recent clinical success of the anti-PD-1 and anti-CTLA-4 immune checkpoint inhibitors has offered an optimistic future for cancer patients. Considerable research effort is now underway to improve the efficacy of immunotherapies. As immune checkpoint inhibitors harness T cells to eradicate cancer cells, the presence of tumor-infiltrating T cells would theoretically improve the efficacy of immunotherapies. A multitude of studies suggested that patients with immunological (or T-cell-infiltrated) tumors respond better to immune checkpoint inhibition as compared with patients with non-immunological (or non-T-cell-infiltrated) tumors^45^. An important pathological study indicated that MDSCs or their derivative TAMs geographically localized in between the margin of CD8^+^ effector T cells and cancer cells, dampening T-cell infiltration^46^. Recent studies indicated that melanoma patients with lower number of M-MDSCs prior treatment responded better to Ipilimumab^47, 48^. These clinical evidences strongly suggested that MDSCs act as a barricade to protect tumors from immunosurveillance. Eradicating MDSCs should enhance the efficacy of cancer immunotherapy. One pioneer study demonstrated that blocking CXCR2 disrupted PMN-MDSC trafficking to tumor sites and sensitized the tumors to anti-PD-1 treatment in mice;^7^ however, this approach specifically targeted PMN-MDSCs but not M-MDSCs and TAMs. Worth mentioning, M-MDSCs are capable to differentiate into PMN-MDSCs in tumor-bearing mice^49^. Therefore, targeting M-MDSCs should be a more preferable therapeutic strategy. Our study identified ENTPD2 as a critical factor in M-MDSC maintenance and we showed that ENTPD2 inhibitor POM-1 suppressed both M-MDSCs and PMN-MDSCs, and sensitized the tumors to anti-PD-1/anti-CTLA-4 immune checkpoint inhibitors.

In summary, our study discloses that hypoxia/HIF-1α induces ENTPD2 in cancer cells to increase the extracellular level of 5′-AMP, which prevents M-MDSCs from maturation, therefore maintaining MDSC undifferentiated in the tumor stroma. Inhibition of ENTPD2 by specific inhibitors can stimulate M-MDSC differentiation into dendritic cell, shifting the stromal cell components from immune suppressive into non-immune suppressive. Our results provide a rationale to devise strategy against MDSCs and increase the efficacy of PD-1/CTLA-4 checkpoint inhibition immunotherapy.

## Methods

### Patient samples and cell lines

Human HCC and their paired NT liver samples were collected during surgical resection at Queen Mary Hospital. The use of human samples was approved by the Institutional Review Board of the University of Hong Kong/ Hospital Authority Hong Kong West Cluster. The patients were explained and they signed consent forms to acknowledge the use of their resected tissues for research purposes. Human HCC cell line MHCC97L was a gift from Fudan University (Dr Z.Y. Tang) of Shanghai. Human HCC cell lines PLC/PRF/5 and Hep3B and murine HCC cell line Hepa1-6 were purchased from American Type Culture Collection. All cell lines were cultured in Dulbecco’s modified Eagle medium (DMEM) supplemented with 10% fetal bovine serum (FBS). Cell cultures were routinely tested for mycoplasma infection.

### Establishment of knockdown, KO and overexpression HCC cells

Mouse Entpd2, ENTPD2, and HIFs knockdown HCC cells were generated by lentiviral-mediated shRNA approach. Oligonucleotides encoding shRNAs targeting ENTPD2, mouse Entpd2, HIF-1α, HIF-2α, non-target control (NTC) were inserted into pLKO.1-puro vector^50^. pLKO plasmids were transfected into HCC cells and selected with puromycin for 7–14 days. HIF-1α-stable KO HCC cell lines were established by TALEN approach. HIF-1α-specific TALE domains were designed by E-TALEN software^51^, assembled and cloned into pTALEN v2 vector by “TALE Toolbox”^52^. HIF-1α KO construct was transfected into MHCC97L HCC cells, respectively. Stable cells were clonally expanded. Frame-shift mutations in the stable KO cells were confirmed by SURVEYOR mutation detection kit (Transgenomic, Inc.) and DNA sequencing. Human ENTPD1/2 and mouse Entpd1/2 stable KO HCC cell lines were established by lentiviral-based CRISPR gene-editing system (lentiCRISPR v2) obtained as a gift from Zhang and colleagues^53^. We sequentially and stably transfected Cas9 and single-guide RNAs (sgRNAs) targeting the first exon of ENTPD1/2 or Entpd1/2 into MHCC97L or Hepa1-6 cells. The KO efficiency was confirmed by flow cytometry. For ENTPD2 overexpressing system, we employ the CRISPR-dCas9 SAM^54^. We sequentially and stably transfected dCas9-VP64, MS2-p65-HSF1, and sgRNAs targeting the promoter of ENTPD2 into MHCC97L cells, as described^54^. shRNA and sgRNA sequences are provided in Supplementary Table 1.

### Antibodies and recombinant proteins

Sources of the antibodies for flow cytometry analysis are listed as follow: mouse Fc Block (1:100; 553142; BD Biosciences), mouse CD45 (1:100; clone 30-F11; Biolegend), mouse Gr1 (1:100; clone RB6-8C5; Biolegend), mouse CD11b (1:100; clone M1/70; eBiosciences), mouse CD11c (1:100; clone N418; Biolegend), mouse F4/80 (1:100; clone MB8; Biolegend), mouse Ly6G (1:100; clone 1A8; Biolegend), mouse Ly6C (1:100; clone HK1.4; Biolegend), human ENTPD2 (1:25; PA5-26333; Sigma-Aldrich), mouse Entpd2 (1:25; ab150503; Abcam), human ENTPD1 (1:100; clone A1; Biolegend), and mouse Entpd1 (1:100; clone Duha59; Biolegend). Sources of the antibodies for immunohistochemistry are listed as follow: human ENTPD2 (1:200; ab150503; Abcam), human GLUT1 (1:1000; ab15309Abcam), and human CA9 (1:500; ab1508L; Abcam).

### Animal studies

For orthotopic implantation, 1 × 10^6^ luciferase-labelled MHCC97L cells were injected into the left lobes of the livers of 5–7-week-old male BALB/c nude mice, whereas 3 × 10^6^ Hepa1-6 cells of C57BL/6 mice. Mice were administered with 100 mg kg^−1^ d-luciferin (Caliper) via intraperitoneal (i.p.) injection before bioluminescent imaging using Xenogen IVIS 100 Imaging System. Tumors were collected for flow cytometry analysis. For drug treatment in orthotopic implantation tumor model, C57BL/6 mice were orthotopically implanted with Hepa1-6 cells and 3 days after the operation were administered with (1) vehicle, 3 mg kg^−1^ ARL67156 (Tocris Bioscience) or 10 mg kg^−1^ POM-1 (Tocris Bioscience) through i.p. injection for 8 consecutive days, and (2) 4 mg kg^−1^ control IgG antibody or 2 mg kg^−1^ PD-1 (clone RMP1-14; BioXCell, West Lebanon, NH) and CTLA-4 (clone 9D9; BioXCell) monoclonal antibodies through i.p. injection on day 5 and day 9. For drug treatment in chemically induced tumor model, 2-week-old C57BL/6 mice were administered with single dose of 1 mg/kg DEN through i.p. injection (Sigma-Aldrich) and after 8 weeks, with 1.25 ml kg^−1^ 30% CCl_4_ (Sigma-Aldrich) through i.p. injection twice weekly for 8 weeks. CCl_4_ (100%) were diluted in olive oil. Twenty-six-week-old mice were with administered 10 mg kg^−1^ POM-1 through i.p. injection thrice weekly or 2 mg kg^−1^ PD-1and CTLA-4 monoclonal antibodies through i.p. injection twice weekly. For in vivo tracing the fate of MDSCs, freshly isolated M-MDSCs or total MDSCs were labelled with 5 µM CFSE dye (Thermo Fisher Scientific). Labeled 5 × 10^5^ M-MDSCs or 1 × 10^6^ total MDSCs were co-injected with 2 × 10^6^ Hepa1-6 cells into the flanks of C57BL/6 mice. Tumors sizes were measured with caliper and calculated with formula: length × width × depth × 0.52 (mm^3^). All animal studies were approved by the Committee on the Use of Live Animals in Teaching and Research, the University of Hong Kong, and performed under the Animals (Control of Experiments) Ordinance of Hong Kong.

### Hypoxia and generation of CM medium

Hypoxic environment was created by culturing cells in 1% O_2_/ 5% CO_2_ in a modulator incubator chamber at 37 ^o^C. To generate CM for MDSC culture, MHCC97L cells were cultured in serum-free DMEM medium and exposed to 20% O_2_ or 1% O_2_ for 24–48 h. Supernatants were collected and purified by 0.20 µm filter. To generate CM for LC-MS, MHCC97L or Hepa1-6 cells were cultured in full medium and exposed to 20% O_2_ or 1% O_2_ for 48 h. Cells were trypsinized and washed with phosphate-buffered saline (PBS). Cells (2 × 10^6^) were re-suspended in serum-free DMEM medium supplemented with 100 µM ATP and incubated at 37 °C for 1 h. Media were collected and purified by 0.20-µm filter.

### Preparation of single cell suspensions

Before magnetic bead cell sorting and flow cytometry analysis, single cell suspensions were prepared. Briefly, spleens and tumors collected from Hepa1-6-bearing C57BL/6 mice were dissociated with gentleMACS dissociator (Miltenyi Biotech) according to the manufacturer’s instruction. Total bone marrow cells were freshly isolated from femurs and tibias of C57BL/6 mice by flushing with PBS. The resulting cell suspensions were filtered through 70 µm cell strainers and treated with ACK lysing buffer (Life Technologies) to remove red blood cells according to the manufacturer’s instruction.

### MDSC isolation by magnetic bead cell sorting

Isolation of CD11b^+^Gr1^+^ total MDSCs, CD11b^+^Ly6C^+^ M-MDSCs, and CD11b^+^Ly6G^+^ PMN-MDSCs were carried out with mouse MDSC isolation kit (Miltenyi Biotech) according to the manufacturer’s instruction. In brief, after treatment with FcR Blocking Reagent, cells were stained with biotin-conjugated Gr1 or Ly6G antibody and further labeled with anti-biotin microbeads. The labeled cells were passed through the MS separation column (Miltenyi Biotech) for magnetic cell separation. The retained cells were analyzed for CD11b^+^Gr^+^/CD11b^+^Ly6C^+^/CD11b^+^Ly6G^+^ population to evaluate for MDSC purity (>90%) by flow cytometry.

### MDSC culture and maintenance

Freshly isolated splenic MDSCs or bone marrow progenitors were cultured in RPMI-1640 medium supplemented with 10% FBS, 10 ng ml^−1^ granulocyte–macrophage colony-stimulating factor (R&D Systems), 10 ng ml^−1^ interleukin-4 (R&D Systems), and 50 µM 2-mercaptoethanol (Sigma-Aldrich), alone or in the presence of 100 µM 5′-AMP (Sigma-Aldrich), 100 µM APCP (Tocris Bioscience), 100 µM adenosine (Sigma-Aldrich), 100 µM NECA (Tocris Bioscience), or HCC cell CM.

### Immunohistochemistry

Before paraffin embedding, human/mouse livers were fixed with 10% formalin and washed with 75% ethanol. Paraffin sections were dewaxed with xylene and rinsed with ethanol. Antigens were retrieved in EDTA buffer by boiling. Sections were stained with primary antibodies overnight at 4 ^o^C and horseradish peroxidase-conjugated secondary antibodies (Dako) for 30 min at room temperature. Sections were developed with 3, 3′-diaminobenzidine (Sigma-Aldrich) and counterstained with hematoxylin. For histological analysis, dewaxed sections were counterstained with hematoxylin and eosin.

### Quantitative real-time PCR

Total RNA was extracted by TRIzol and complementary DNA was prepared using GeneAmp Gold RNA PCR Core Kit (Applied Biosystems). Quantitative real-time PCR amplification of ENTPD2 and the internal control HPRT were performed using the Taqman Gene Expression Assay for clinical specimens and using SYBR Green qPCR Master Mix (Applied Biosystems) with specific primers provided in Supplementary Table 2 for other expression studies.

### Clinicopathological correlation

The mRNA level of ENTPD2 in HCC patients was correlated with different clinicopathological parameters in HCC patients by SPSS20.0 software (SPSS, Inc.)^56^. Briefly, the parameters were analyzed by pathologist upon surgical resection. The clinicopathological parameters included venous invasion, direct liver invasion, tumor size, absence of tumor encapsulation, presence of tumor microsatellite formation, cellular differentiation by Edmondson grading, and association with hepatitis B infection. Clinicopathological correlations of ENTPD2 expression were performed with Student’s *t*-test. Survival test was done by Kaplan–Meier curve and log-rank test. *P* < 0.05 was considered statistically significant.

### Flow cytometry analysis

Cells were incubated with anti-mouse Fc Block for 10 min at room temperature and stained with fluorophore-conjugated antibodies for 45 min at 4 ^o^C in staining buffer (PBS with 0.5% bovine serum albumin, and 2 mM EDTA). Labeled cells were washed with staining buffer twice and analyzed by BD FACSCantoII Analyzer (BD Biosciences) and FlowJo software (FlowJo). Staining condition of other dyes for functional assays were described individually.

### HCC cell proliferation assay

The proliferation rate of HCC cell line was measured by cell counting. For both MHCC97L and Hepa1-6 subclones, 2 × 10^4^ cells were seeded on 12-well culture plates and cultured in 20 and 1% O_2_ for 4 days. Cells were trypsinized and counted with Z1 coulter particle counter (Beckman Coulter) daily.

### T-cell proliferation assay

Isolation of CD4^+^CD3^+^ T cells was carried out with mouse T-cell isolation kit (R&D Systems) according to the manufacturer’s instruction. Isolated T cells were stained with 2 µM CSFE for 10 min and subsequently co-cultured with freshly isolated CD11b^+^Gr1^+^ MDSCs in different ratios (1:1; 2:1; 4:1, and 1:0) for 3 days. The fluorescence intensity of CFSE for each cell would halve after cell division. Number of cell division of each cell was analyzed by flow cytometry.

### ChIP assay

MHCC-97L cells were crosslinked with formaldehyde, lysed with SDS buffer and sonicated. Sheared DNA was precleared with salmon sperm DNA/protein A agarose slurry (Merck Millipore) and immunoprecipitated with HIF-1α, HIF-1β, and IgG (Santa Cruz). Agarose beads were incubated with antibody/protein/DNA complex and washed with low-salt buffer, high-salt buffer, and LiCl wash buffer according to the manufacturer’s protocol (Millipore). DNA was eluted in 1% SDS/0.1 M NaHCO_3_ and de-crosslinked with 0.2 M NaCl.

### ATP and 5′-AMP measurements

The amounts of ATP, 5′-AMP and adenosine in CM were quantified by liquid chromatography–tandem mass spectrometry (LC-MS). CM from cells were collected and mixed with methanol. For LC-MS analysis, the metabolites were reconstituted in 50% methanol (v/v). The chromatographic separation was performed in a hypercarbon analytical column at a flow rate of 200 µl min^−1^. The samples were separated using a mobile phase consisting of 10 mM ammonium acetate in water (solvent A) and in acetonitrile (solvent B) that were adjusted with ammonia to pH 10.0. The gradient elution was performed in 98% solvent A decreased to 50% solvent A in 10 min and then back to 98% solvent A in 10 min. Eluted metabolites were analyzed in the multiple reaction monitoring (MRM) mode using 3200 QTRAP System (Applied Biosystems Sciex). Standard of 5′-AMP and ATP (Sigma) was used for optimization. The MRM transition of 5′-AMP was set *m*/*z* 345.95/134.10 and ATP was set *m*/*z* 506.00/159.00. The amount of 5′-AMP was represented by the area of analyst peak.

### Statistical analysis

Data are expressed as mean ± SD and analyzed by Student’s *t*-test. *P*-values <0.05 were considered significant.

### Data availability

The TCGA data reference ion this study are available in the cBioPortal for Cancer Genomics website http://www.cbioportal.org/. The authors declare that all the other data supporting the findings of this study are available within the article and its Supplementary Information files and from the corresponding author upon reasonable request.

## Electronic supplementary material

Supplementary Information
