# Supplementary material for: Hypoxia inducible factor HIF-1 promotes myeloid-derived suppressor cells accumulation through ENTPD2/CD39L1 in hepatocellular carcinoma
Source: Nat Commun. 2017 Sep 11;8:517. doi: 10.1038/s41467-017-00530-7 (PMC5593860; doi:10.1038/s41467-017-00530-7)
Supplement: Supplementary file 1 — Supplementary Information [file 41467_2017_530_MOESM1_ESM.pdf]

Description of Supplementary Files

File Name: Supplementary Information
Description: Supplementary Figures and Supplementary Tables

a

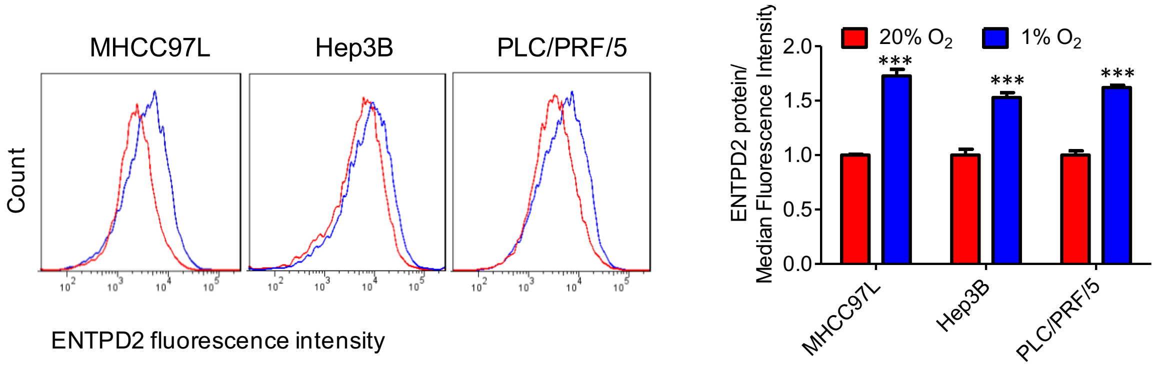

b

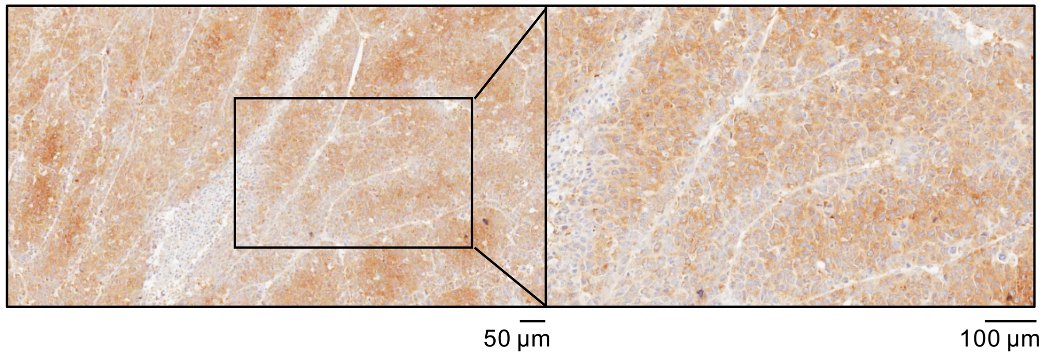

Supplementary figure 1. The protein expression of ENTPD2 in HCC cell lines and tissues. (a) The protein expressions of ENTPD2 in MHCC97L, Hep3B and PLC/PRF/5 exposed to 20% and 1% O_2 for 48 hr were determined by flow cytometry (n = 3). **(b)** Immunohistochemical staining of ENTPD2 in human HCC tissues displayed a patchy pattern which is a typical oxygen diffusion pattern. Data are presented as mean±s.d. (Student's t-test, *** *P*<0.001)

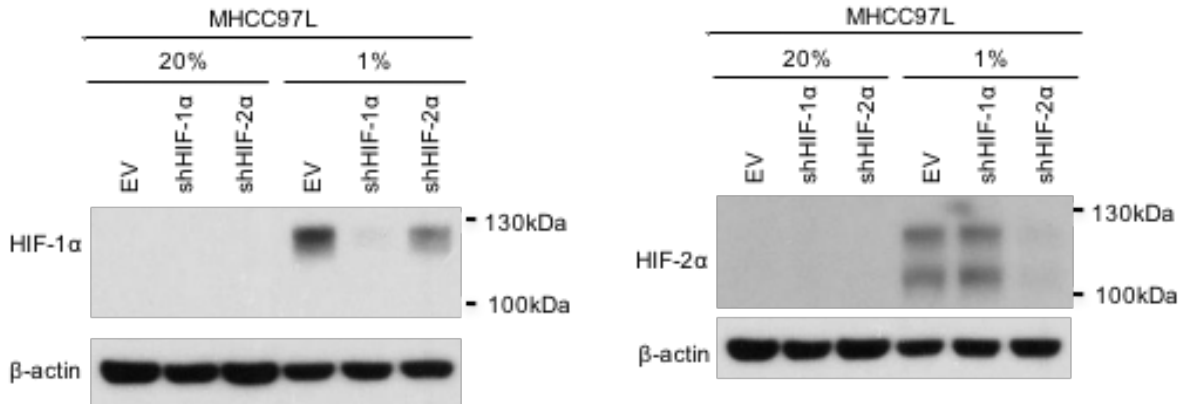

Supplementary figure 2. The knockdown efficiency of HIF-1α and HIF-2β in MHCC97L confirmed by western blot. HIF-1α and HIF-2α protein expressions in MHCC97L-EV, -shHIF-1α, -shHIF-2α cells that were exposed to 20% and 1% O_2 for 4 hours.

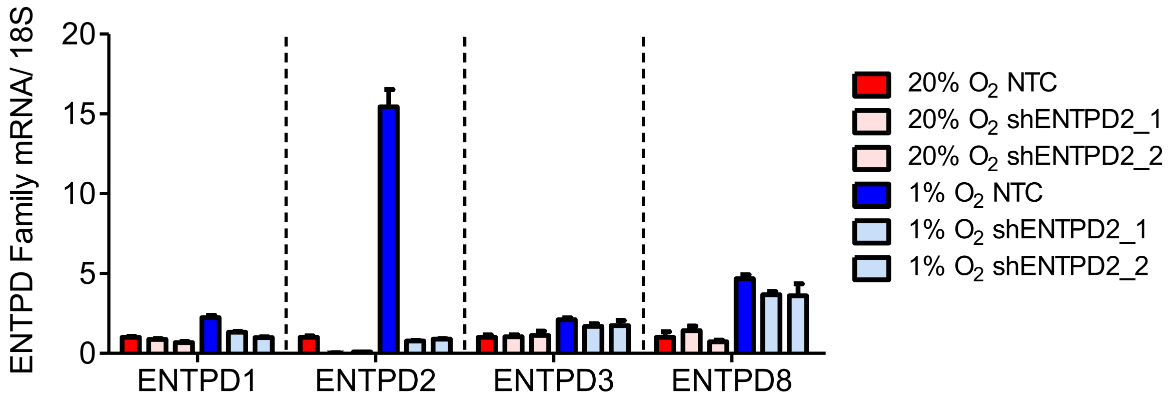

Supplementary figure 3. The mRNA expression of CD39 family in ENTPD2 knockdown HCC cells. The mRNA expressions of ENTPD1, ENTPD2, ENTPD3 and ENTPD8 in MHCC97L-NTC and –shENTPD2 clones. Cell were exposed to 20% and 1% O_2 for 24 hr (n = 3). qRT-PCR values were normalized to 20% O_2. Data are presented as mean±s.d.

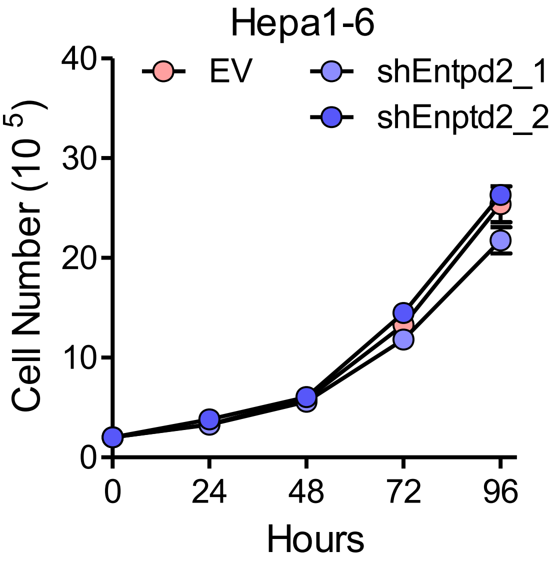

Supplementary figure 4. Proliferation rate of Hepa1-6-EV and -shEntpd2 clones (n = 3). Data are presented as mean±s.d. No statistically significant difference was found between the proliferation of Hepa1-6-EV and –shEntpd2 clones. (Student's t-test, P>0.05)

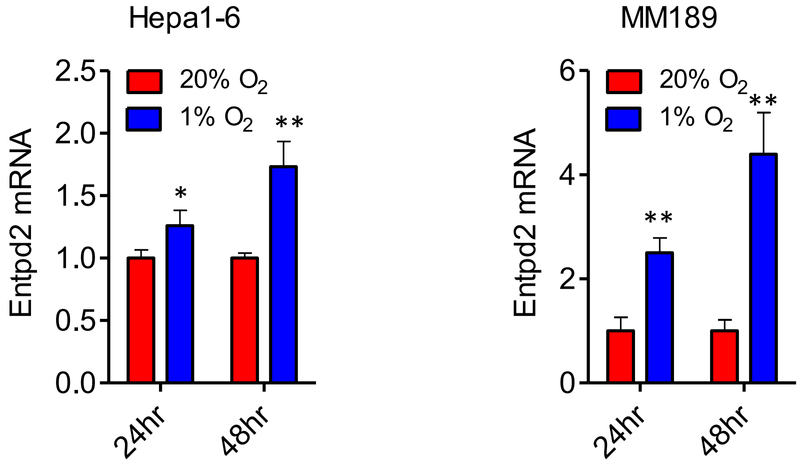

Supplementary figure 5. The mRNA expression of Entpd2 in mouse HCC cell lines. The mRNA expressions of Entpd2 in Hepa1-6 and MM189. Cells were exposed to 20% and 1% O_2 for 24-48 hr (n = 3). qRT-PCR values were normalized to 20% O_2. Data are presented as mean±s.d. (Student's t-test, * P<0.05, ** P<0.01)

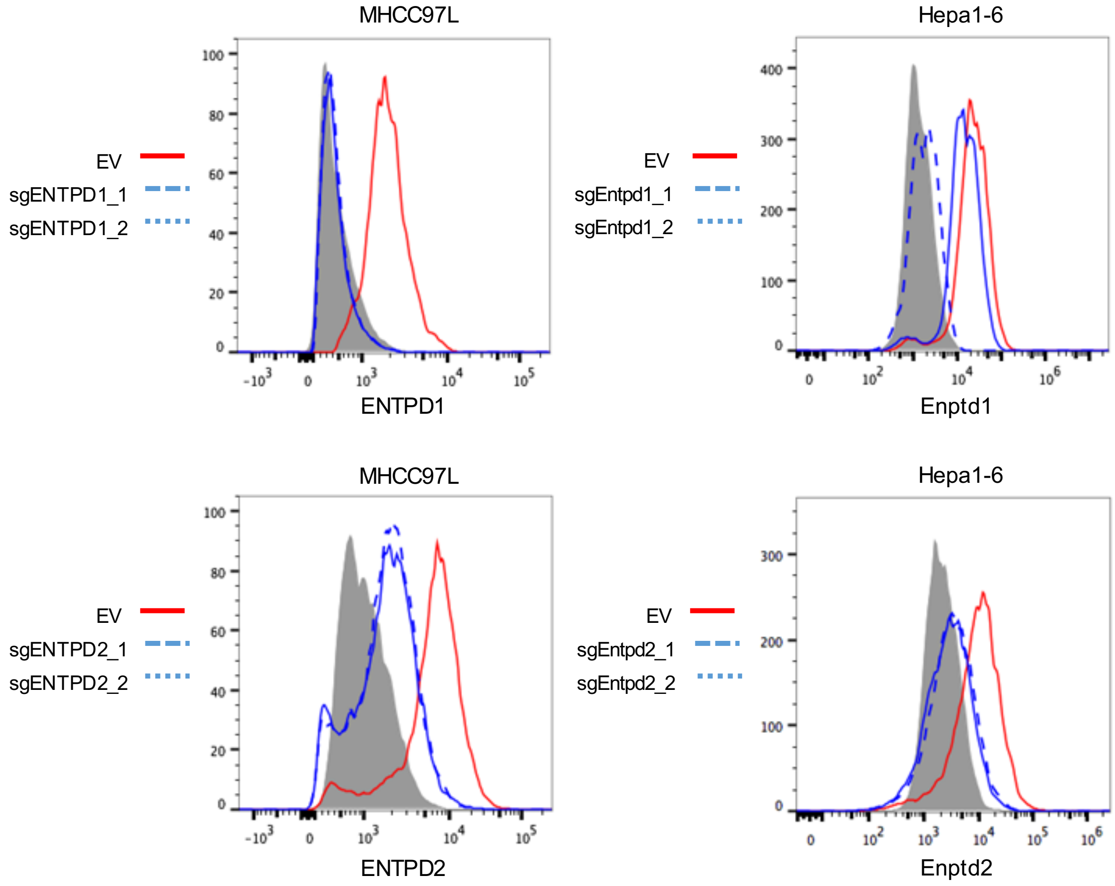

Supplementary figure 6. Establishment of MHCC97L-sgENTPD1/2 and Hepa1-6-sgEntpd1/2 clones. Protein expressions of ENTPD1/2 in MHCC97L and Entpd1/2 in Hepa1-6 clones were determined by flow cytometry (n = 3). Grey area indicated the control isotype.

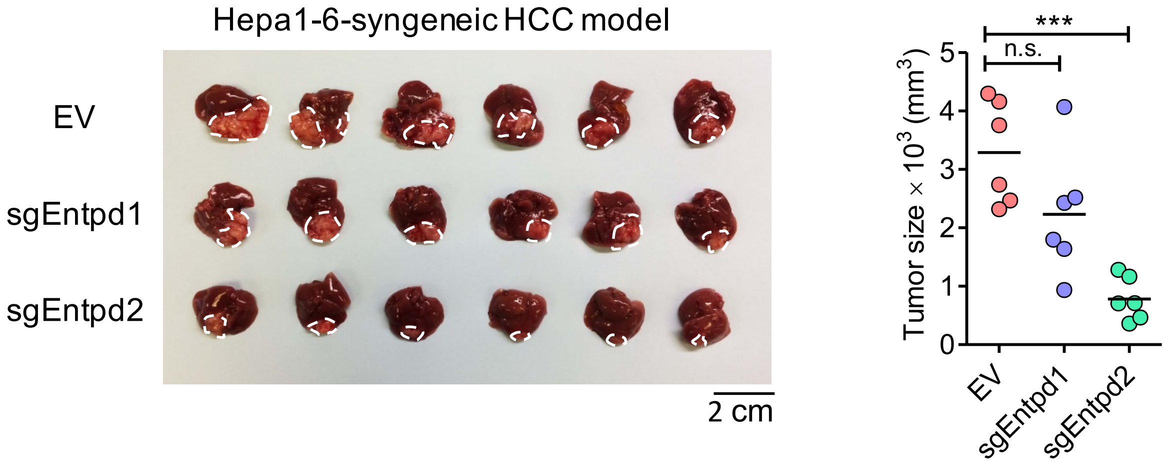

Supplementary figure 7. The effects of ENTPD1 and ENTPD2 on HCC growth. C57BL/6 mice were orthotopically implanted with 3×10^{6} Hepa1-6-EV, -sgEntpd1 and -sgEntpd2 (n = 6 for each group). Tumor size was measured with caliper. (Student's t-test, *** P<0.001)

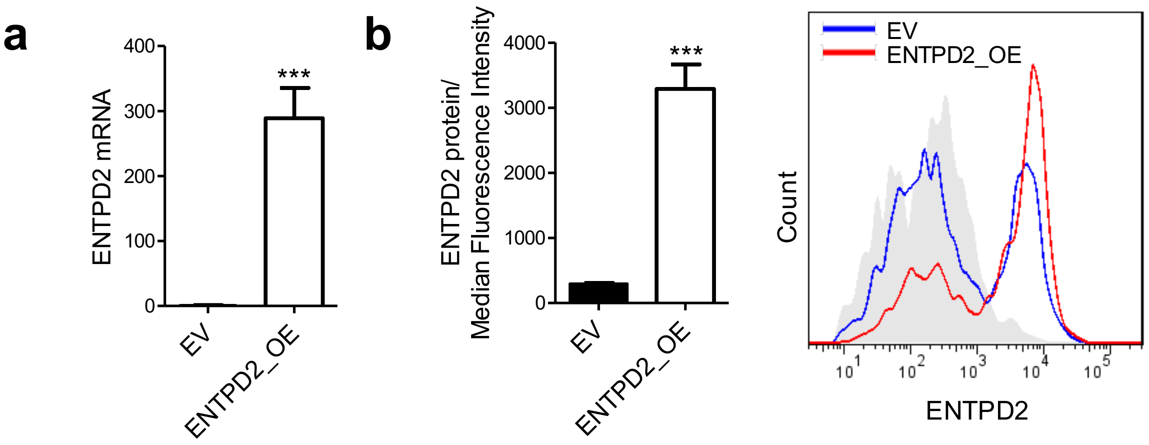

Supplementary figure 8. Establishment of MHCC97L-ENTPD2 overexpressing (OE) clones. (a) mRNA and **(b)** protein expressions of ENTPD2 in MHCC97L-EV and -ENTPD2-OE clones were determined by qRT-PCR and flow cytometry respectively (n = 3). Grey area indicated the control isotype. The expressions were normalized to EV. Data are presented as mean±s.d. (Student's t-test, *** P<0.001)

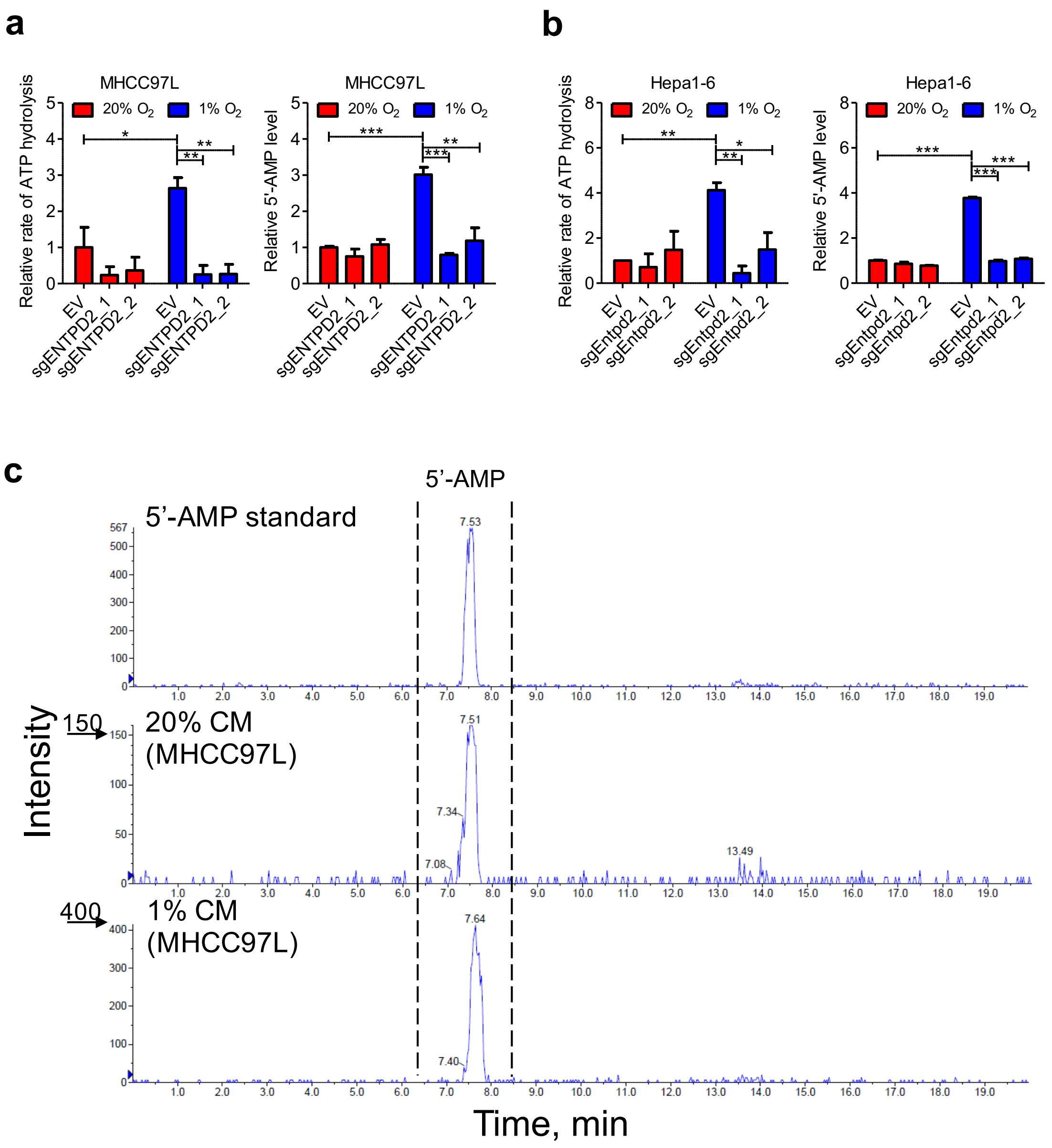

Supplementary figure 9. The effects of ENTPD2-knockout on extracellular ATP hydrolysis and 5'-AMP production in HCC cell lines. (a) MHCC97L and **(b)** Hepa1-6 cells were pre-exposed to 20%O_2 and 1%O_2 for 48 hr. Cells were trypsinized and 2×10^6 cells were re-suspended in serum-free DMEM medium supplemented with 100 µM ATP and incubated at 37 ^{o}C for 1 hr. CM were collected and subjected to LC-MS analysis (n = 3). **(c)** Representative peaks of 5'-AMP in 5'-AMP standard solution, and CM from MHCC97L cells exposed to 20%O_2 and 1%O_2. Data are presented as mean±s.d. (Student's t-test, * P<0.05, ** P<0.01, *** P<0.001)

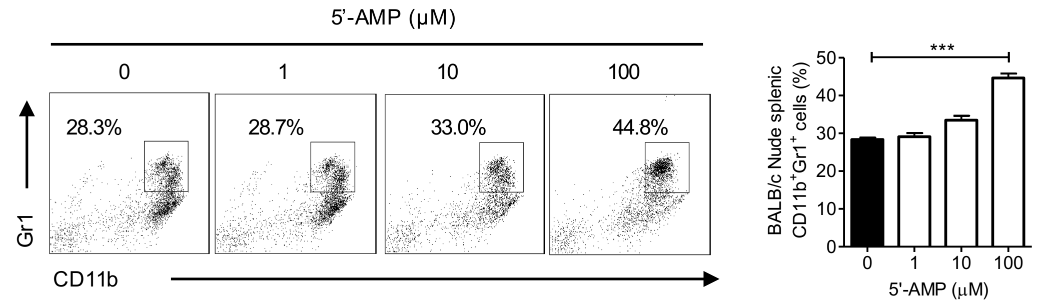

Supplementary figure 10. The effect of 5'-AMP on total MDSC maintenance. Splenic $CD11b^{+}Gr1^{+}$ cells isolated from BALB/c nude mice were cultured in the presence of different concentrations of 5'-AMP. The percentages of $CD11b^{+}Gr1^{+}$ cells after 4-day culturing were analyzed (n = 3). Data are presented as mean±s.d. (Student's t-test, *** $P<0.001$)

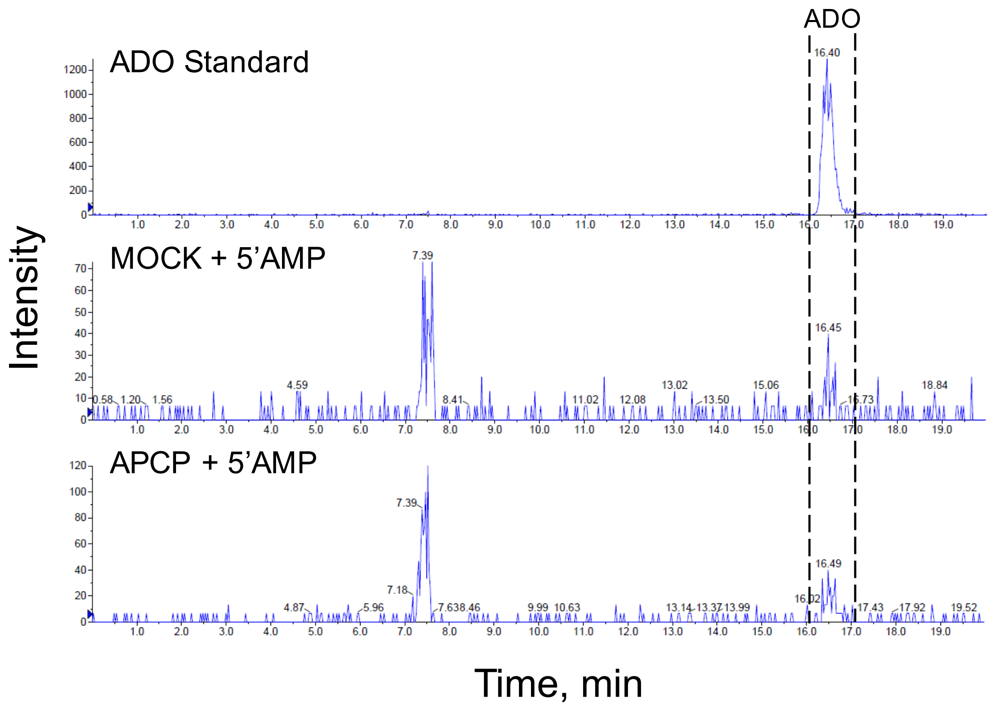

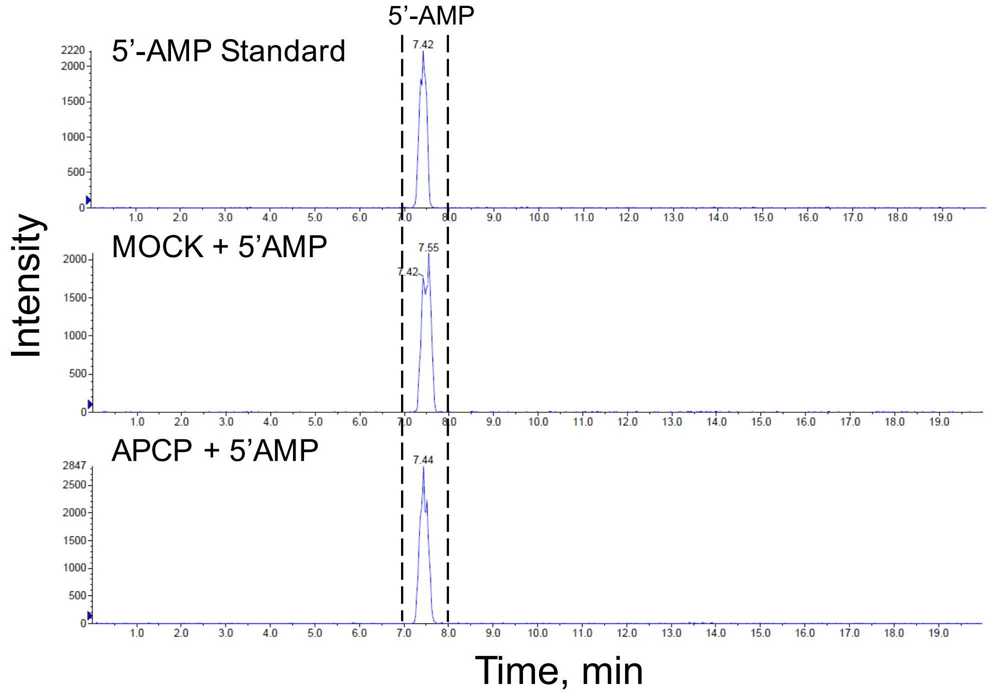

Supplementary figure 11. The level of 5'-AMP in MDSC culture. The effect of 5'-AMP on MDSC maintenance. Splenic $CD11b^{+}Gr1^{+}$ cells isolated from C57BL/6 mice were cultured in the presence of 100 μM 5'-AMP with or without NT5E inhibitor, APCP. CM were collected after 24 hr and subjected to LC-MS analysis (n = 3). The peaks representing 5'-AMP and adenosine (ADO) in 5'-AMP/ADO standard solution and CM were shown.

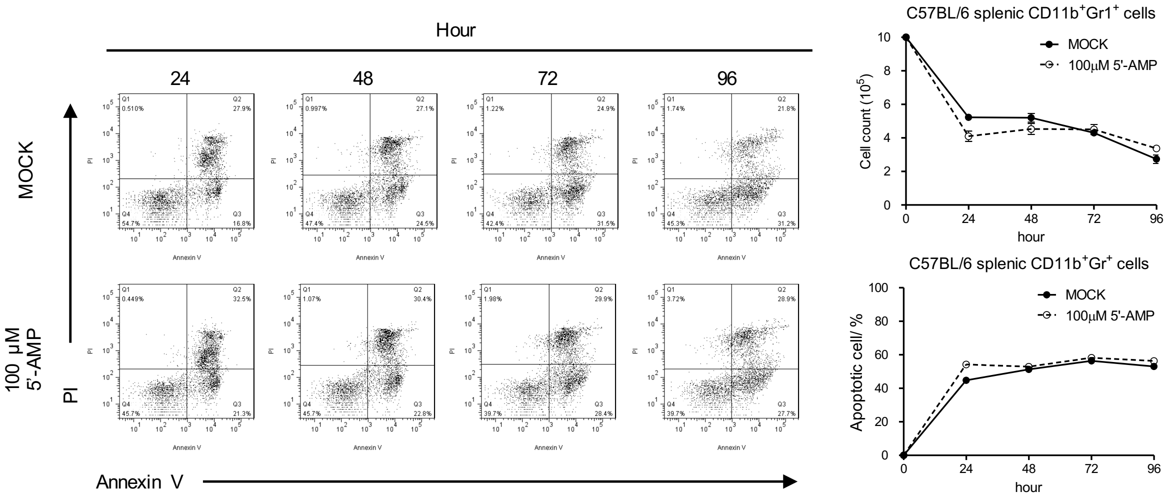

Supplementary figure 12. The effect of 5'-AMP on the apoptotic rate of total MDSCs. Splenic $CD11b^{+}Gr1^{+}$ cells isolated from C57BL/6 mice were cultured with or without 100 μM 5'-AMP. Annexin V and propidium iodide (PI) staining was performed in cells at different time points. Percentage of apoptotic cells was measured by flow cytometry analysis (n = 3). Data are presented as mean±s.d. No statistically significant difference was found. (Student's t-test, P>0.05)

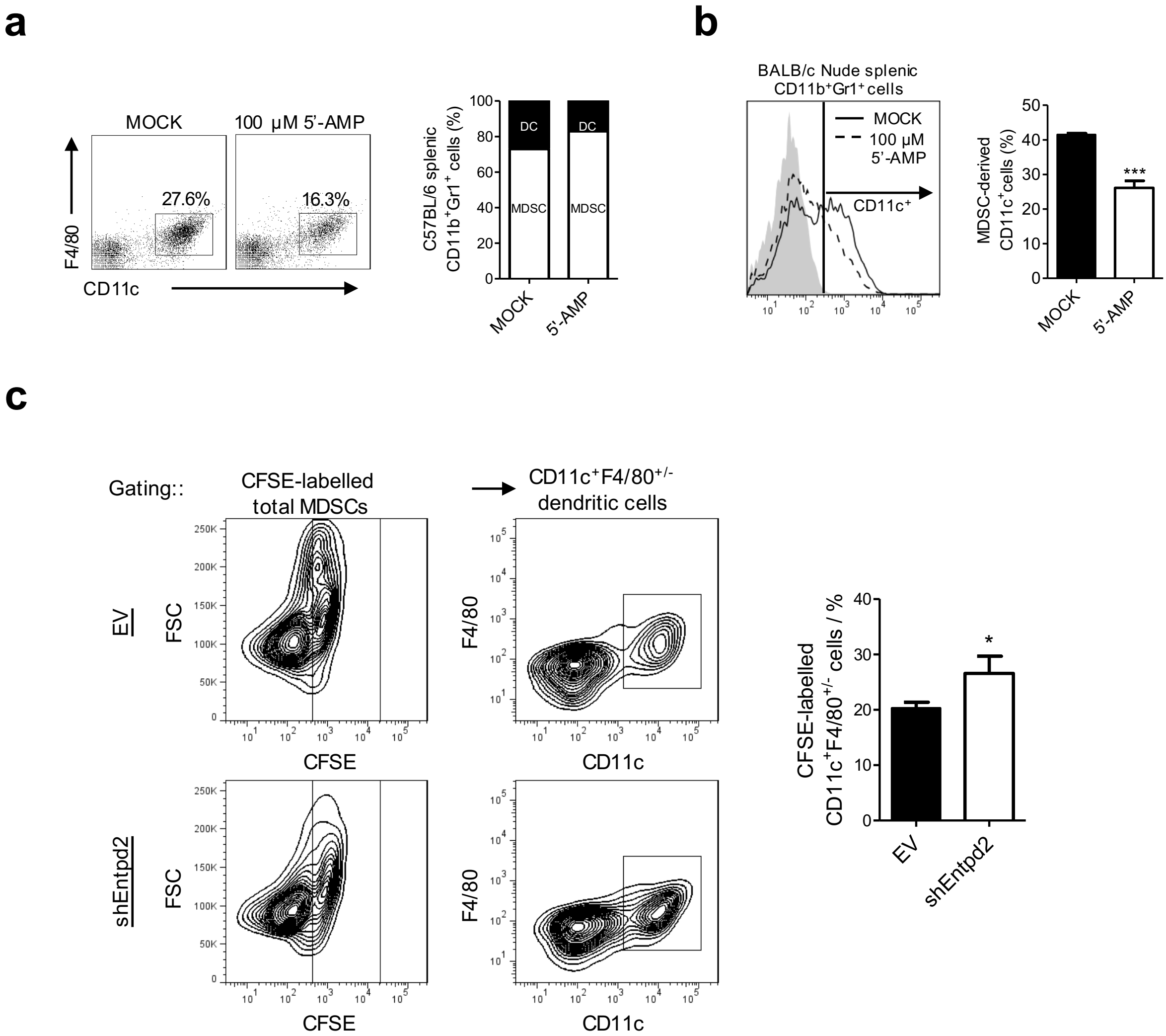

Supplementary figure 13. The effect of 5'-AMP on total MDSC differentiation. Splenic CD11b^{+}Gr1^{+} cells isolated from **(a)** C57BL/6 mice or **(b)** BALB/c nude mice were cultured with or without 100 µM 5'-AMP for 4 days. The distribution of dendritic cell and total MDSC populations were determined by the surface marker CD11c by flow cytometry (n = 3). Grey area indicated the control isotype. **(c)** The effect of ENTPD2 on the fate of total MDSCs *in vivo*. Splenic CD11b^{+}Gr1^{+} cells isolated from C57BL/6 mice were stained with fluorescent cell tracking dye CFSE and co-inoculated with Hepa1-6-EV or shEntpd2 clones into C57BL/6 mice subcutaneously (n = 3 for each groups). The tumors were dissociated and stained with anti-CD11c and anti-F4/80 to examine the differentiation of CFSE-labelled total MDSCs. Data are presented as mean±s.d. (Student's t-test, * P<0.05, *** P<0.001)

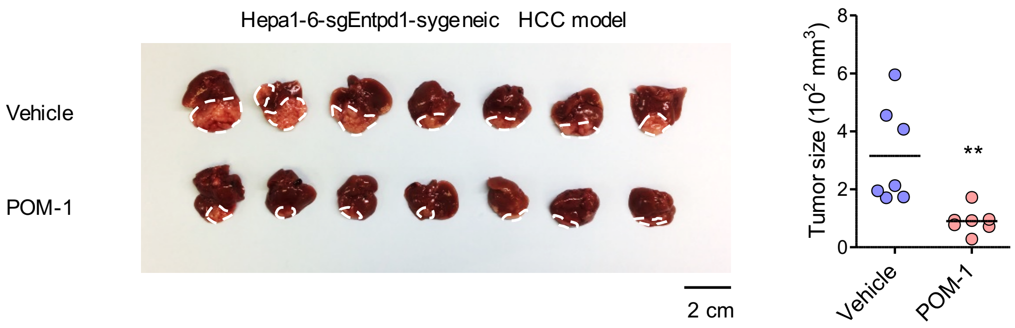

Supplementary figure 14. The effect of POM-1 on Hepa1-6-sgEntpd1-derived tumors. C57BL/6 mice were orthotopically implanted with 3×10^6 Hepa1-6-sgEntpd1. On day 3, mice were administered with vehicle or 10 mg/kg POM-1 through i.p. injection for 8 consecutive days (n = 7 for each group). Images of tumors harvested from mice and tumor size was measured with caliper. (Student's t-test, ** P<0.01)

Supplementary Table 1 | Target sequences of shRNAs and sgRNAs

sh/sgRNA clones	Gene Bank	Target nucleotides
Human shENTPD2	NM_001246	150-170
Mouse shEntpd2_1	NM_009849	529-549
Mouse shEntpd2_2	NM_009849	629-649
Human shHIF-1α	NM_181054	2,123-2,141
Human shHIF-2α	NM_001430	1,992-2,012
Human sg-ENTPD2 (activation)	NM_001246	CGGCGGAAGCTTGGGAGCGG
Human sg-ENTPD2_1	NM_001246	GCGTCCCCACCCGCGACGTC
Human sg-ENTPD2_2	NM_001246	GCTCCCGGACGTCGCGGGTG
Human sg-ENTPD1_1	NM_001776	GTTGATAGTAATCCAGCCAT
Human sg-ENTPD1_2	NM_001776	CTCTTGGCCAGTAATGATCC
Mouse sg-Entpd2_1	NM_009849	CGGGCGGCTCCCGGACGTCT
Mouse sg-Entpd2_2	NM_009849	GGCTCCCGGACGTCTTGGGT
Mouse sg-Entpd1_1	NM_001304721	GAGCTATCACAGCCAAGATAG
Mouse sg-Entpd1_2	NM_001304721	CCTTGGTTTCACCTCTATCT

Supplementary Table 2 | Primer sequences

Genes	Sequences (5'-3')
Human ENTPD1	Forward: CAGGGGGATTTTGGGGCATT
	Reverse: TACTTCTCCTTTACTCCAGCGT
Human ENTPD2	Forward: ACCCACAGCTTCCTCTGCTA
	Reverse: CTGAAGAGCCCAGAAACCAG
Human ENTPD3	Forward: CCATTTGTGGCTTTTGCAGGA
	Reverse: CTGACTCCAATTCTGTGAGCA
Human ENTPD4	Forward: AGCGAAGCCATTGTCCGTAA
	Reverse: ACTTCTTCCTGCTGTGAGGAC
Human ENTPD5	Forward: GACAGCACAGTCTTACAGCTCA
	Reverse: CAATGGGAGATGCCCAGAGAC
Human ENTPD6	Forward: TTCGAGATCGCAGCCAAGTA
	Reverse: CCGAGTGAGCTTCAGCACTT
Human ENTPD7	Forward: ATGCAGAACAGCAGGATCAGT
	Reverse: AGAAGAGTCCCAGGAATGCCA
Human ENTPD8	Forward: CACAGTTGAAGGGACAGGCA
	Reverse: GCTGGCCTCCACATAGAACT
Human NT5E	Forward: ATGAACGCAACAATGGCACAA
	Reverse: CCTTCTTCAGGGTGGAACCTT
Human 18S	Forward: GAGGATGAGGTGGAACGTGT
	Reverse: AGAAGTGACGCAGCCCTCTA
Mouse Entpd2	Forward: GCCCTCAAGTATGGCATCGT
	Reverse: CGAACATCGCAAGAGCTGTG
Mouse 18s	Forward: ACATCGACCTCACCAAGAGG
	Reverse: TCCCATCCTTCACATCCTTC

Human ENTPD2-317 (ChIP)	Forward: CTCCACAAACAGGTGCTGAC<br>Reverse: CGTGGAGGCAGGAGACCT
Human ENTPD2-2215 (ChIP)	Forward: CCACAACCGGCTAATTTTTG<br>Reverse: TGCCTCAGTCTCCACATCAG
Human ENTPD2-2497 (ChIP)	Forward: GGGTTCACACCATTCTCCTG<br>Reverse: CGGTGGCTCACTCCTGTAAT
